# High malaria transmission in a forested malaria focus in French Guiana: How can exophagic *Anopheles darlingi* thwart vector control and prevention measures?

**DOI:** 10.1590/0074-02760160150

**Published:** 2016-09

**Authors:** Samuel B Vezenegho, Antoine Adde, Vincent Pommier de Santi, Jean Issaly, Romuald Carinci, Pascal Gaborit, Isabelle Dusfour, Romain Girod, Sébastien Briolant

**Affiliations:** 1 Institut Pasteur de la Guyane, Unité d’Entomologie Médicale, Cayenne, Guyane, France; 2 Centre d’Epidémiologie et de Santé Publique des Armées, Marseille, France; 3 Direction Interarmées du Service de Santé en Guyane, Cayenne, Guyane, France; 4 Institut de Recherche Biomédicale des Armées, Département des Maladies Infectieuses, Unité de Parasitologie et d’Entomologie, Brétigny sur Orge, France; 5 Aix Marseille Université, Unité de Recherche sur les Maladies Infectieuses et Tropicales Emergentes, Marseille, France

**Keywords:** Anopheles darling, Plasmodium vivax, biting behaviour, entomological inoculation rate, malaria, French Guiana

## Abstract

In French Guiana, malaria vector control and prevention relies on indoor residual spraying and distribution of long lasting insecticidal nets. These measures are based on solid epidemiological evidence but reveal a poor understanding of the vector. The current study investigated the behaviour of both vectors and humans in relation to the ongoing prevention strategies. In 2012 and 2013, *Anopheles* mosquitoes were sampled outdoors at different seasons and in various time slots. The collected mosquitoes were identified and screened for *Plasmodium* infection. Data on human behaviour and malaria episodes were obtained from an interview. A total of 3,135 *Anopheles* mosquitoes were collected, of which *Anopheles darlingi* was the predominant species (96.2%). For the December 2012-February 2013 period, the *Plasmodium vivax* infection rate for *An. darlingi* was 7.8%, and the entomological inoculation rate was 35.7 infective bites per person per three-month span. In spite of high bednet usage (95.7%) in 2012 and 2013, 52.2% and 37.0% of the participants, respectively, had at least one malaria episode. *An. darlingi* displayed heterogeneous biting behaviour that peaked between 20:30 and 22:30; however, 27.6% of the inhabitants were not yet protected by bednets by 21:30. The use of additional individual and collective protective measures is required to limit exposure to infective mosquito bites and reduce vector densities.

Malaria in French Guiana, an overseas territory of France located on the northeast coast of South America, remains of public health importance even though the number of reported clinical cases has dropped from 4,479 cases in 2005 to 445 cases in 2014 (Ardillon et al. 2015). Since 2005, *Plasmodium vivax* has been predominant; this species was responsible for 67% of the reported malaria cases in the territory in 2014, the other cases were mainly due to *P. falciparum* (Carme et al. 2009, Musset et al. 2014, Ardillon et al. 2015). Most of the officially reported cases occur in villages located along the main rivers that flow through the territory. Moreover, it is clear that illegal gold mining areas are obviously relevant uncontrolled locations for malaria transmission (Pommier de Santi et al. 2016a, b, c). The coastal region of the territory is home to 75% of the population and is essentially characterised by imported malaria cases from the inland areas, although autochthonous transmission is occasionally observed (Carme et al. 2009, Musset et al. 2014, Ardillon et al. 2015).

A total of 24 anopheline species have been reported in French Guiana (Talaga et al. 2015). One of these is *Anopheles (Nyssorhynchus) darlingi*, known as the most common malaria vector in the Americas and, consequently, also in the Amazon Region (Sinka et al. 2010, Hiwat & Bretas 2011, Laporta et al. 2015). This species has been incriminated as the principal malaria vector in French Guiana at different periods and places (Floch & Abonnenc 1951, Floch 1955, Girod et al. 2008, Hiwat et al. 2009, Fouque et al. 2010, Girod et al. 2011). Furthermore, other species (*An. nuneztovari sl*, *An. oswaldoi sl*, *An. intermedius*, *An. marajoara* and *An. ininii*) that have been found naturally infected with *Plasmodium* sporozoites in the wild are considered as secondary vectors, although their role in human transmission is still to be unravelled (Dusfour et al. 2012, Pommier de Santi et al. 2016a, b).

Malaria vector control in French Guiana is achieved through the distribution of long-lasting insecticidal nets (LLINs) and indoor residual spraying (IRS). Deltamethrin is the insecticide used for both approaches (CIRE 2006, Mansotte et al. 2010). Whereas IRS targets malaria vectors that rest inside houses, LLINs prevent contact between humans and mosquitoes, which often bite indoors when people are sleeping. The deltamethrin used in both strategies is supposed to protect the community by killing the vectors and interrupting local malaria transmission.

The effectiveness of LLINs is reduced when mosquitoes change their biting behaviour from times when LLINs users are under their nets to when they are not using them. In the same way, the effectiveness of IRS is reduced when mosquitoes adopt exophagic and exophilic behaviours.

Indeed, *An. darlingi* exhibits heterogeneous biting behaviour across its range. Indoor biting by this species has been reported in Belize (Roberts et al. 2002), while a tendency to bite outdoors has been noted elsewhere, including Brazil (Gil et al. 2003), Suriname (Rozendaal 1989, Hiwat et al. 2011) and Peru (Moreno et al. 2015). In French Guiana, this species has been collected while biting humans outside houses in both the coastal region and in forested areas (Pajot et al. 1977, Girod et al. 2008, Dusfour et al. 2010, 2013, Vezenegho et al. 2014, 2015). These entomological observations should be considered in the light of human behaviour to define an efficient and sustainable malaria vector control strategy. In particular, special attention should be given to the outdoor activities of residents from soon after sunset until the later part of the evening when they retire into their houses. The same issues may arise at dawn.

Beyond *Anopheles* human biting rates, the longevity of the female population is a key parameter in estimating the risk of malaria transmission. This risk can be assessed by measuring the parity of females (Detinova 1962). Indeed, older females are more likely to transmit the parasite given the length of its sporogonic cycle. However, data on the longevity of *An. darlingi* populations are very scarce in French Guiana where parity is generally low (Girod et al. 2008, Hiwat et al. 2009, Fouque et al. 2010).

The success of malaria vector control strategies is reflected by a decrease in malaria transmission. Several indices such as parasite rate, annual parasite index, spleen rate and entomological inoculation rate can be used to measure malaria transmission (Hay et al. 2000, Smith & McKenzie 2004, Smith et al. 2007, 20, 2009). The entomological inoculation rate (EIR) is considered the most direct of these indices and provides an insight into the impact of malaria control measures. Moreover, it helps to identify areas of increased risk. The strength of EIR lies in the fact that it quantifies the parasite-infected mosquitoes and their probability of transmitting parasites to humans.

With the goal of maintaining the recent gains that have reduced the number of malaria cases in the territory, an entomological study was conducted in Blondin, a village located along the Oyapock River separating French Guiana from Brazil. The objectives were (i) to assess the biting behaviour of *Anopheles* mosquitoes and their role in malaria transmission, and (ii) to characterise human behaviour and investigate malarial episodes in relation to the ongoing malaria vector control and prevention strategies.

## MATERIALS AND METHODS

**Fig. 1:**
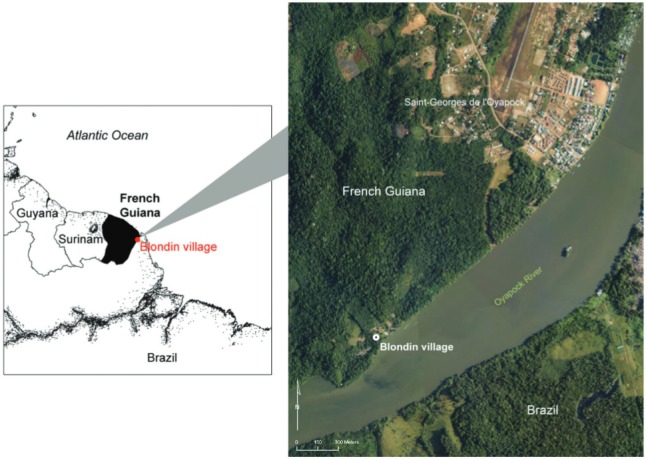
: localisation of the study site of Blondin village, Saint-Georges de l’Oyapock municipality, French Guiana. Orthophotograph acquired in 2006 by the French National Geographic Institute (BD-ORTHO® product).

*Study site* - The study was conducted in Blondin, French Guiana, a forested village located along the Oyapock River, which forms a natural boundary with Brazil, upstream of the town of Saint-Georges de l’Oyapock. A health centre is available to the municipality, which is occupied by 3,855 inhabitants (Fig. 1). Blondin village itself is home to approximately 60 inhabitants, primarily Amerindians and people of Brazilian origin. The village comprises 13 houses, which have wooden walls and corrugated aluminium roofs without ceilings. Inhabitants of the village engage in subsistent farming and fishing.

The climate of the study area is equatorial, and four seasons of unequal length can be distinguished: a long rainy season from April to June, a long dry season from July to December, a short rainy season from January to February and a short dry season in March. The short dry and wet seasons are yearly periods of low and high rainfall, respectively. The transition periods are the yearly intervals between these low and high rainfall periods. In French Guiana, the seasons are influenced by the migration of the inter-tropical convergence zone. In 2012 and 2013, the average annual rainfall was 3126.5 mm and 3131.1 mm, respectively. During the study period, from December 2012 to February 2013 (session 1) and from September to November 2013 (session 2), average rainfall was 891.8 mm and 304.9 mm, respectively. The average temperature is approximately 27ºC with an average relative humidity of 80%. In French Guiana, the average annual rainfall is approximately 3,000 millimetres.

Malaria in the village is mainly due to *P. vivax*. An increased incidence of cases is usually observed between October and December. Distribution of LLINs in Blondin village started in July 2012 with a coverage level of 100%. Each year, IRS is implemented in September - before the major peak in malaria cases.

In the municipality of Saint-Georges de l’Oyapock, 257 and 152 malaria cases were diagnosed in 2012 and 2013, respectively. *P. vivax* and *P. falciparum* were, respectively, responsible for 88.7% and 11.3% of malaria cases in 2012, and for 90.8% and 9.2% of malaria cases in 2013.

*Study design and mosquito collection* - Mosquito collections were conducted in Blondin village from December 2012 to February 2013 (session 1) and from September 2013 to November 2013 (session 2). Mosquitoes were collected for four and three consecutive nights in the first week of each month for sessions 1 and 2, respectively. The collections were performed from 18:30 to 20:30, 20:30 to 22:30 and 05:00 to 07:00 by human landing catches (HLC). The collection sites were the same throughout the study. Mosquitoes were collected exclusively outdoors and close to houses. HLC were performed by two individuals, each acting as bait and collector following World Health Organization recommendations, whereby the lower legs are exposed and landing mosquitoes collected with a mouth aspirator (WHO 1975).

*Field and laboratory processing* - *Anopheline* species were identified morphologically using taxonomic keys specific to the region (Floch & Abonnenc 1951, Forattini 1962, Faran & Linthicum 1981). A subset of at most ten *An. darlingi* per day was dissected for determination of parity status by examining the conformation of tracheoles (Detinova 1962).

*Genomic DNA extraction and Plasmodium species identification* - DNA was extracted from the head and thorax of all female anopheline mosquitoes according to the protocol provided by the MagMAX^TM^-96 DNA Multi-Sample kit (Applied Biosystems, USA). The resultant DNA in pools of ten was subjected to nested polymerase chain reaction (PCR) to detect *Plasmodium* DNA according to Snounou et al. (1993). Individually stocked DNA used to compose the pooled DNA was screened when a pool tested positive.

*Survey interview* - The interviews were conducted during the dry season in 2014 to avoid seasonal bias in the answers. The interview first explored socio-demographic characteristics such as age and gender, duration of residence in Blondin village and level of education. Participants were then asked whether they knew the role of mosquitoes in malaria transmission, the signs of malaria in adults and children and were questioned about the use of preventive measures (nets and repellents), their behaviour (the time they retire under bednets to sleep and the time they emerge from the bednets), and malaria history (episodes in 2012 and 2013, whether they had consulted a doctor and whether they took anti-malarial drugs without instructions from a doctor). Interviews were performed in the presence of a translator for assistance in situations involving language barriers.

*Data and statistical analysis* - Human biting rates (HBR) were calculated as the total number of anopheline mosquitoes caught landing on humans, divided by the number of collectors, divided by the number of hours spent sampling. *Plasmodium* species infection rates were calculated as the number of specimens infected with the *Plasmodium* parasite divided by the total number of specimens tested. The entomological inoculation rate estimates the number of infective mosquito bites per person per unit of time. It is calculated as the product of the HBR and the infection rate (Birley & Charlewood 1987).

All statistical analyses were performed with GraphPad Prism software (version 5.01). Quantitative variables were compared using Mann-Whitney tests. Percentages were compared by using Fisher’s exact test. The comparison of *Anopheles* distributions between session 1 and 2 was performed by using Chi-square tests. The comparison of *Anopheles* distributions according to the collection time within a session was performed by using Chi-square tests for trend. Statistical results were considered significant when the p-value was below 0.05.

*Ethics* - The collectors were local volunteer residents who were given training on HLC and informed of the associated risks of the collection method. They were supervised during the captures by the authors. Malaria prophylaxis was proposed and information on the medication was provided. Collectors who benefited from prophylaxis gave their free, express and informed consent. The interviews were anonymous and completed with the consent of all participants.

## RESULTS

*Anopheles species composition and human biting rates* - A total of 3,135 *Anopheles* mosquitoes were collected (Table I). During session 1,274 specimens were collected and 95.6% [CI95% (92.5-97.7)] of the *Anopheles* mosquitoes were identified as belonging to four species. *An. darlingi* was the predominant species [89.0%, CI95% (84.7-92.5)] followed by *An. nuneztovari sl* [4.4%, CI95% (2.3-7.5)], *An. triannulatus sl* [1.8%, CI95% (0.6-4.2)] and *An. braziliensis* [0.4%, CI95% (0.0-2.0)]. During session 2, 2,861 *Anopheles* mosquitoes were collected and 97.2% [CI95% (96.6-97.8)] were identified as belonging to three species. *An. darlingi* was again the predominant species [96.9%, CI95% (96.2-97.5)], followed by *An. nuneztovari sl* [0.2%, CI95% (0.1-0.5)] and *An. braziliensis* [0.1%, CI95% (0.0-0.2)]. The difference in the distribution of *Anopheles* species between sessions 1 and 2 was statistically significant (p < 0.0001, Chi-square test). Regarding HBR, *An. darlingi* HBR was significantly higher than for other *Anopheles* species in both sessions (p < 0.0001, Fisher’s exact test). The HBR for *An. darlingi* was significantly higher during session 2 than during session 1 (p < 0.0001, Fisher’s exact test).

**TABLE I:** Distribution and human biting rates (HBR) of *Anopheles* species collected in Blondin village, Saint-Georges de l’Oyapock municipality, French Guiana, using human landing catches from December 2012 to February 2013 (session 1) and September to November 2013 (session 2)

Collection session	Species	Number (%)	Total hours of collection	HBR (number of bites/human/hour)
1	*An. sp*	12 (4.4%)	144	0.042
	*An. braziliensis*	1 (0.4%)	144	0.003
	*An. darlingi*	244 (89.0%)	144	0.847
	*An. nuneztovari s.l.*	12 (4.4%)	144	0.042
	*An. triannulatus s.l.*	5 (1.8%)	144	0.017
	Total	274 (100%)	144	0.951
2	*An. sp*	79 (2.8%)	108	0.366
	*An. braziliensis*	2 (0.1%)	108	0.009
	*An. darlingi*	2,773 (96.9%)	108	12.838
	*An. nuneztovari s.l.*	7 (0.2%)	108	0.032
	Total	2,861 (100%)	108	13.245

**Fig. 2:**
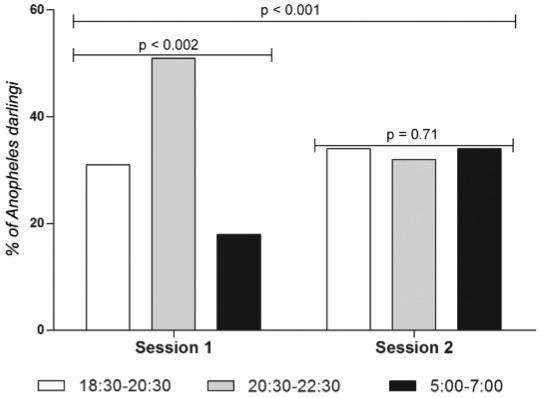
: percentage of *Anopheles darlingi* by collection time slot in Blondin village, Saint-Georges de l’Oyapock municipality, French Guiana, December 2012 to February 2013 (session 1) and September to November 2013 (session 2).

*An. darlingi distribution and HBR by collection time* - The distribution of *An. darlingi* by number and HBR according to the collection time and session are detailed in Table II. The distribution of *An. darlingi* density per collection time (Fig. 2) was significantly different between session 1 and 2 (p < 0.001, Chi-square test). There was no difference between the percentages of *An. darlingi* collected regarding the time slots (p = 0.71, Chi-square test for trend) for session 1; however, there was a significant difference between the percentages of *An. darlingi* regarding the collection time in session 2 (p = 0.002, Chi-square test for trend). The percentage of *An. darlingi* [50.8%, CI95% (44.4-57.3)] caught between 20:30 and 22:30 was significantly higher than those caught between 18:30 and 20:30 [31.2%, CI95% (25.4-37.4)] and between 05:00 and 7:00 [18.0%, CI95% (13.4-23.4)] respectively; p < 0.0001, Fisher’s exact test.

**TABLE II:** *Anopheles darlingi* distribution and human biting rates (HBR) by collection time slots in Blondin village, Saint-Georges de l’Oyapock municipality, French Guiana, using human landing catches from December 2012 to February 2013 (session 1) and September to November 2013 (session 2)

Collection session	Collection period	Number (%)	Total hours of collection	HBR (number of bites/human/hour)
1	18:30-20:30	76 (31.2%)	46	0.264
	20:30-22:30	124 (50.8%)	46	0.430
	05:00-07:00	44 (18.0%)	46	0.153
	Total	244 (100%)	144	0.847
2	18:30-20:30	934 (33.7%)	36	4.324
	20:30-22:30	892 (32.2%)	36	4.130
	05:00-07:00	947 (34.1%)	36	4.384
	Total	2,773 (100%)	108	12.838

*An. darlingi infection rate, entomological inoculation rate and parity* - None of the *Anopheles* mosquitoes collected during either session was infected with *P. falciparum*, and *An. darlingi* was the only species found infected with *P. vivax* during session 1, with an infection rate (IR) of 7.8% [n = 19/244, CI95% (4.8-11.9)]. There was no difference IR of *An. darlingi* with regard to the collection time (p = 0.81, Chi-square test for trend). From 18:30 to 20:30, *An. darlingi* IR was 6.6%, CI95% (2.2-14.7); from 20:30 to 22:30, it was 8.9%, CI95% (4.5-15.3); and from 05:00 to 07:00, it was 6.8%, CI95% (1.4-18.7). A global EIR of 0.07 infective bites per person per hour was obtained for *An. darlingi* during session 1. Considering that people were bitten during six hours per night (being under bednets the rest of the night), to estimate the level of malaria transmission during the high transmission risk period, a global three-months EIR was calculated, resulting in 35.7 infective bites per person per three months [CI95% (25.0-49.4)]. However, none of the mosquitoes collected in session 2 was infected with *P. vivax*.

**Fig. 3:**
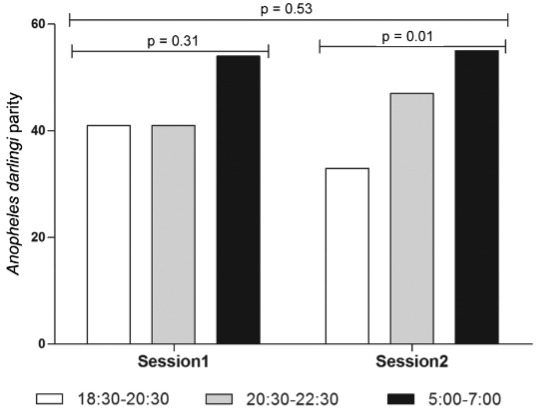
: dynamics in *Anopheles darlingi* parity in Blondin village, Saint-Georges de l’Oyapock municipality, French Guiana, December 2012 to February 2013 (session 1) and September to November 2013 (session 2).

The parity per collection time obtained for a total of 315 *An. darlingi* females (106 and 209, respectively, for sessions 1 and 2) are presented in Fig. 3 and Table III.

**TABLE III:** *Anopheles darlingi* parity by collection time slot in Blondin village, Saint-Georges de l’Oyapock municipality, French Guiana, using human landing catches from December 2012 to February 2013 (session 1) and September to November 2013 (session 2)

Session	Collection period	Number	Parous	Non parous	Undetermined	Parity (CI95%)
1	18:30-20:30	76	20	29	0	40.8 (27.0-55.8)
	20:30-22:30	124	12	17	0	41.4 (23.5-61.1)
	05:00-07:00	44	15	13	0	53.6 (33.9-72.5)
	Total	244	47	59	0	44.3 (34.9-53.8)
2	18:30-20:30	934	21	43	2	32.8 (21.6-45.7)
	20:30-22:30	892	33	37	2	47.1 (35.1-59.4)
	05:00-07:00	947	41	34	1	54.7 (42.8-66.2)
	Total	2,773	95	114	5	45.5 (38.6-52.5)

There was no difference between the distributions of *An. darlingi* parity with regard to the collection time in session 1 and 2 (p = 0.53, Chi-square test), and during session 1, there was no difference in *An. darlingi* parity according to the time slot of collection (p = 0.31, Chi-square test for trend). However, during session 2, there was a significant difference in *An. darlingi* parity between the time slot of collection (p = 0.01, Chi-square test for trend). The *An. darlingi* parity assessed from 05:00 to 07:00 [54.7%, CI95% (42.7-66.2)] was significantly higher (p = 0.01, Fisher’s exact test) than the *An. darlingi* parity assessed from 18:30 to 20:30 [32.8%, CI95% (21.6-45.7)].

**Fig. 4:**
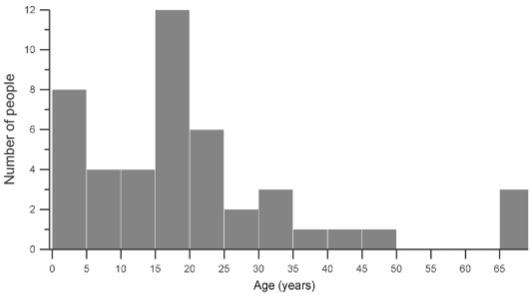
: age distribution of inhabitants in Blondin village.

*Survey interview* - Among 60 inhabitants in Blondin village, 46 were questioned - 26 males (56.5%) and 20 females (43.5%) whose median age was 19 years (min = six months, max = 71 years). The distribution of Blondin village residents by age class (period of five years) is represented in Fig. 4. The majority of the participants (93.5%, n = 43) have lived in the village since its creation or from birth. The rest of the participants (6.5%, n = 3) arrived in the village the year preceding the study.

In total, 95.7% (n = 44) of the respondents acknowledged using LLINs each night when they went to bed. The mean hour at which participants went to bed was 21:14 [CI95% (20:53-21:35)] and the mean hour at which they arose was 6:44 [CI95% (6:26-7:02)]. Only three participants (6.5%) used repellents for personal protection against mosquito bites.

**Fig. 5:**
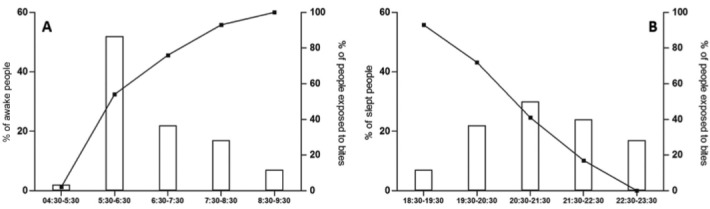
: (A) percentages of people awake (bars) and exposed to *Anopheles darlingi* bites (curves) by time slot; (B) percentages of people sleeping (bars) and exposed to *An. darlingi* bites (curves) by time slot.

Distributions of awake and sleeping people by time slot and the cumulative percentage of people exposed to *An. darlingi* bites are shown in Fig. 5.

**Fig. 6:**
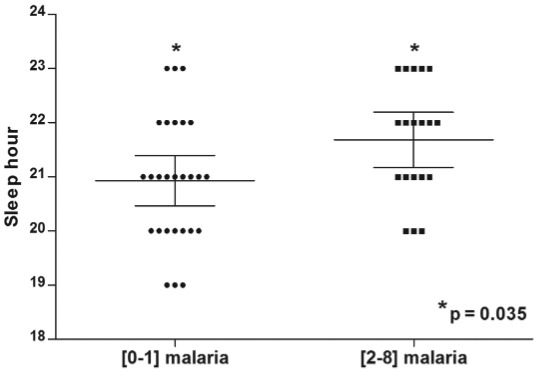
: comparison of hours at which people retire between those who suffered (0-1) and (2-8) malaria episodes during 2012 and 2013, respectively, in Blondin village, Saint-Georges de l’Oyapock municipality, French Guiana.

At the time of survey, 52.2% (n = 24) and 37.0% (n = 17) of the participants acknowledged having contracted malaria at least once during 2012-2013 study period. As shown in Fig. 6, the median bedtime hour (22:00, 25% percentile of 21:00 and 75% percentile of 23:00) for people who suffered more than two malaria episodes during 2012 and 2013 was significantly higher than the median (21:00, 25% percentile at 20:00 and 75% percentile at 22:00) in people who had no or only one malaria episode (p = 0.03, Mann Whitney test).

**Fig. 7:**
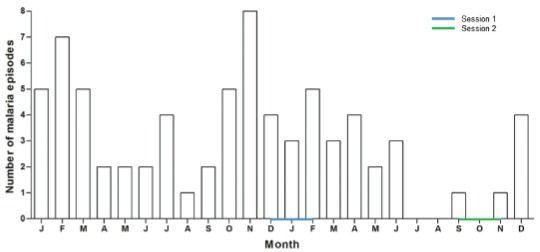
: evolution of malaria episodes by participants during the years 2012 and 2013 in Blondin village, Saint-Georges de l’Oyapock municipality, French Guiana.

Among the people who suffered from malaria episodes in 2012 (n = 24), 12.5% (n = 3) were below five years old and 87.5% (n = 21) were above five years old. Participants who had malaria in 2013 represented 37.0% (n = 17). Of these, 5.9% (n = 1) were below five years old and 94.1% (n = 16) were above five years old. Fig. 7 shows the variation in malaria episodes for the years 2012 and 2013 according to participants. During 2012, malaria episodes were more numerous from January to March and then from October to December. In 2013, malaria episodes essentially occurred from January to April and in December.

## DISCUSSION

The current study was designed to unravel malaria transmission mechanisms and to determine whether the currently implemented malaria vector control strategies are tailored to the behaviour of the main vectors and human inhabitants. The study was carried out in Blondin Village, in the municipality of Saint-Georges de l’Oyapock, one of several areas in French Guiana where residual malaria transmission still challenges the control programme instituted by health authorities. The data collected in this study indicate that *An. darlingi* is predominant of the four anopheline species present. This observation is in accord with the findings of other studies carried out in the territory (Girod et al. 2008, 2011, Hiwat et al. 2009, Fouque et al. 2010, Dusfour et al. 2012). *An. darlingi* was significantly more abundant during session 2, which corresponded to the long dry season in French Guiana. Similar situations have already been observed in other areas in Amazonia (Gil et al. 2003). However, the peak abundance of this species has been more frequently reported in the rainy season in French Guiana (Magris et al. 2007, Girod et al. 2008, 2011, Hiwat et al. 2009, Fouque et al. 2010) as well as in other areas (da Silva-Vasconcelos et al. 2002, de Barros & Honorio 2007, Moreno et al. 2007). Because sampling during session 2 started in the middle of the long dry season, the observed abundance can be attributed to the availability of large residual water collections left behind from precipitation during the preceding long rainy season and/or floods as the river progressively retreated to its lowest level. These breeding sites result in mass production of adult mosquitoes at the beginning of the long dry season, generating high densities of adult mosquitoes during the second part of the long dry season. Sampling performed during session 1 coincided with the short rainy season just after the long dry season. The end of the long dry season is characterised by a decrease in flooding of the Oyapock River which, together with the limited rainfall, resulted in few breeding sites and, consequently, the low number of *An. darlingi* collected.

To investigate the role of anopheline species in malaria transmission in the village, collected mosquitoes were screened for both *P. vivax* and *P. falciparum* infection. *An. darlingi* collected during session 1 was the only species infected with *P. vivax*. The involvement of *An. darlingi* in malaria transmission is well documented in South America, especially in French Guiana (Girod et al. 2008, 2011, Hiwat et al. 2009, Fouque et al. 2010). Unexpectedly, no specimens from session 2 were infected with *Plasmodium* species. One possible explanation is that interview responses indicated that malaria cases reached zero prior to mosquito collection during the second session. In contrast, session 1 followed a period during which the number of reported malaria cases was much more significant, which may explain the observed infection rates. In the current study, the global three-month EIR estimating the level of malaria transmission during the high-risk period was calculated during session 1 and based on six hours of mosquito bites exposures per night. This three-month EIR corresponds to 35.7 infective bites/person/three months [CI95% (25.0-49.4)], which is high compared to annual EIR for other parts of French Guiana such as the Upper-Maroni area with a range of 14.4 to 27.4 infective bites/person/year (Girod et al. 2008), Apatou with 5.7 infective bites/person/year, Regina with 8.7 infective bites/person/year (Girod et al. 2011), Loca with 10.0 infective bites/person/year and Twenke with 5.0 infective bites/person/year (Fouque et al. 2010). Although the time units used to calculate the EIR in these studies differs, the present data clearly suggest that Blondin village is a high-risk area for contracting malaria.

The behaviour of both *An. darlingi* and humans in Blondin village were studied to gather baseline data to assist in realigning current malaria control strategies. The biting behaviour of *An. darlingi* reached a peak from 20:30-22:30 in session 1; however, during session 2, there was no statistically significant difference between the percentages of *An. darlingi* collected during the three time slots. First, this can be attributed to the high number of *An. darlingi* landing simultaneously on humans’ legs, generating a saturation effect during the collections. Second, it has already been reported that the biting behaviour of *An. darlingi* can differ based on the season (Charlwood 1996, Voorham 2002, Leon et al. 2003, Zimmerman et al. 2013). The evening peak biting time in session 1 corresponded to the village residents’ mean bedtime; however, prior to this time, biting activity (which began as soon as 18:30) progressively increased while the majority of the people were still awake and exposed to mosquito bites. Similarly, early in the morning, although the biting rate is low, most of the village inhabitants are out of bed and exposed to *An. darlingi* bites. Moreover, parity being more important in the morning, inhabitants are exposed to the bites of older females, increasing the likelihood of malaria transmission. Finally, considering the number of malaria cases reported by residents and their sleeping hours, it is clear that people not protected by bednets at the beginning of the night and early in the morning are at high risk of contracting malaria.

Inhabitants exposed to infective mosquito bites during these periods should adopt additional personal protection measures such as cutaneous repellents and mosquito coils and should wear clothing - if possible, impregnated with insecticide - that covers the body. Interestingly, most of the inhabitants did not use any of these personal protective measures.

The structure of houses in this area might also bear some responsibility in malaria transmission. The village’s wooden-walled houses had gaps that may allow mosquitoes to move in and out. Coupled with the lack of ceilings these structures provide favourable mosquito environments and contribute to the occupants’ risk of contracting malaria. In this situation, the use of LLINs and IRS are essential to protect inhabitants - but not sufficient. French Guiana is truly in urgent need of study regarding the effectiveness of IRS and the indoor resting behaviour of *An. darlingi*. In this work, IRS was carried out in September, before the peak of transmission. Nevertheless the data showed a peak in the number of cases in October. Due to location of the houses relative to potential breeding sites and the evident exophilic and exophagic behaviour of *An. darlingi*, it would be interesting to develop complementary strategies to protect people. For example, using “pull and push” or “lure and kill” approaches employing attractant-baited traps and repellents or toxic compounds or screens impregnated with insecticides placed between the resting and breeding sites and the houses could constitute a realistic option considering the size and configuration of the village (Burkot et al. 2013, Matowo et al. 2013, Menger et al. 2015).

Malaria vector control strategies should be aligned with the behaviours of both the malaria vectors and the human population to be protected. *An. darlingi*, the dominant of four *Anopheles* species identified in an area of residual malaria transmission in French Guiana, has been incriminated as the main vector of *P. vivax*. In the studied village, inhabitants’ outdoor activities just after dusk and at dawn favour exposure to *An. darlingi* infective bites. Consequently, even though LLINs coverage in the village exceeds the Roll Back Malaria target of 80% and IRS are applied just before the peak transmission period, these control strategies do not provide complete protection. This study recommends the use of individual protective measures and also highlights the need to test innovative collective measures to protect people against outdoor bites from *Anopheles*.
